# Toward Single-Atomic-Layer Lithography on Highly Oriented Pyrolytic Graphite Surfaces Using AFM-Based Electrochemical Etching

**DOI:** 10.1007/s41871-022-00127-9

**Published:** 2022-03-11

**Authors:** Wei Han, Paven Thomas Mathew, Srikanth Kolagatla, Brian J. Rodriguez, Fengzhou Fang

**Affiliations:** 1 Centre of Micro/Nano Manufacturing Technology (MNMT-Dublin), University College Dublin, Dublin, D04 V1W8 Ireland; 2 Shanghai Engineering Research Centre of Ultra-Precision Optical Manufacturing, Fudan University, Shanghai, 200433 China; 3 School of Physics, University College Dublin, Dublin, D04 V1W8 Ireland; 4 Conway Institute of Biomolecular and Biomedical Research, University College Dublin, Dublin, D04 V1W8 Ireland; 5 State Key Laboratory of Precision Measuring Technology and Instruments, Laboratory of Micro/Nano Manufacturing Technology (MNMT), Tianjin University, Tianjin, 300072 China

**Keywords:** Etching, Lithography, Electrochemical machining, Atomic and close-to-atomic scale manufacturing (ACSM)

## Abstract

Atomic force microscopy (AFM)-based electrochemical etching of a highly oriented pyrolytic graphite (HOPG) surface is studied toward the single-atomic-layer lithography of intricate patterns. Electrochemical etching is performed in the water meniscus formed between the AFM tip apex and HOPG surface due to a capillary effect under controlled high relative humidity (~ 75%) at otherwise ambient conditions. The conditions to etch nano-holes, nano-lines, and other intricate patterns are investigated. The electrochemical reactions of HOPG etching should not generate debris due to the conversion of graphite to gaseous CO and CO_2_ based on etching reactions. However, debris is observed on the etched HOPG surface, and incomplete gasification of carbon occurs during the etching process, resulting in the generation of solid intermediates. Moreover, the applied potential is of critical importance for precise etching, and the precision is also significantly influenced by the AFM tip wear. This study shows that the AFM-based electrochemical etching has the potential to remove the material in a single-atomic-layer precision. This result is likely because the etching process is based on anodic dissolution, resulting in the material removal atom by atom.

## Introduction

Nanomanufacturing involves scaled-up, reliable, and cost-effective manufacturing of nanoscale materials, structures, devices, and systems [1]. It leads to the production of improved materials and new products, and manufactured structures with unique properties in the nanoscale are capable of enabling quantum leaps and improvement in high-performance technologies, from new sensors, high-density data storage, and drug delivery to high-strength materials and energy-efficient solar cells [2, 3]. These applications lead to significant demand in the future research and development of nanomanufacturing. Based on material properties, nanomanufacturing can be performed by additive, subtractive, and mass conservation. Several nanomanufacturing technologies, such as laser ablation [4], etching [5], ultraviolet light lithography [6], and focused ion beam (FIB) [7], have been widely utilized to obtain functional structures and surfaces with nanoscale features. Although exciting results have been achieved, many challenges are still encountered in nanomanufacturing relative to the nanoscale, nano accuracy, complex shape/structure, and novel materials [2, 8].

Scanning probe microscopy (SPM)-based lithography, as a promising nanolithography approach for the fabrication at the nanometer scale, has attracted significant attention because of its simplicity and precise control of a structure and location [9]. The development of scanning tunneling microscopy (STM) has facilitated the research in STM-based nanomanufacturing, considering that STM tip-induced surface local oxidation was found on a measured sample surface [10]. However, atomic force microscopy (AFM), as a kind of SPM, shows more advantages in nanomanufacturing than STM, especially because AFM can work in ambient environments. Moreover, many different approaches, such as chemical [11] and electrical [12] methods, can be easily combined on AFM to improve the nanomanufacturing ability. AFM-based electrochemical machining was first used to modify hydrogen-passivated *n*-Si(111) surfaces via chemical oxidization in ambient conditions [11]. Accordingly, the kinetics and mechanism of oxidation have attracted great interest due to the major contributions in the machining process [13, 14], and other papers have hoped to improve the reproducibility of the process by studying the dynamic force microscopy modes [15, 16]. The comprehensive understanding and control of the oxidation mechanism are of critical importance for the application of the SPM technique. However, the complexity of challenges remains open, and the oxidation process of the sample is still complicated [17]. Furthermore, atomic and close-to-atomic scale manufacturing (ACSM) has become the leading trend in global manufacturing development [3, 18]. To achieve ACSM, AFM and STM work as vital instruments due to the atomic and close-to-atomic scale resolution in all three spatial dimensions. For decades, scientists have been inspired to develop relevant techniques to ACSM to directly visualize and manipulate an individual atom using SPM [19–21].

**Fig. 1:**
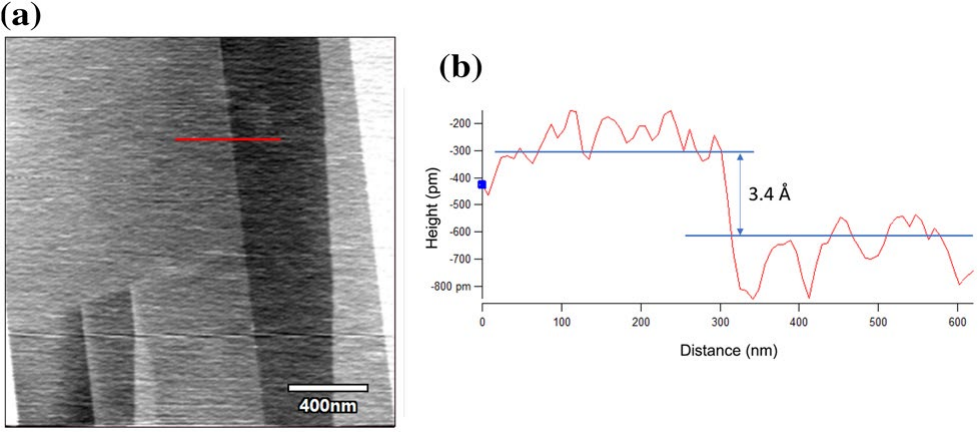
Step thickness obtained for a single layer of HOPG: **a** AFM image of a single step edge of the HOPG surface. **b** Line profile showing a thickness of 3.4 Å between two layers

Highly oriented pyrolytic graphite (HOPG) is a highly ordered form of high-purity pyrolytic graphite, a graphite material with a high degree of preferred crystallographic orientation. HOPG is typically obtained via the graphitization heat treatment of carbon or chemical vapor deposition under pressure and high temperatures. It is usually used in nanolithography and obtaining graphene due to the ease of preparation of atomically flat surfaces and layered structures. The thickness of single-layer graphene is 3.35 Å [22, 23], and the expected single layer of material removal is less than 5 Å. The step thickness obtained for a single layer of HOPG is shown in Fig. 1.

In this study, an AFM-based electrochemical machining method was used to etch the HOPG surface to single-atomic-layer precision. Compared with previous studies that nano-dots and nano-holes were widely fabricated [24–26], the material removal toward single-atomic-layer was focused, and intricate patterns were to be etched. As a preliminary study, nano-holes and nano-lines were etched to investigate the material removal process first. Then, intricate patterns were etched on the HOPG surface toward single-atomic-layer precision. Because of the trend of ACSM in global manufacturing, this study also explored the capability of the AFM-based electrochemical machining technique in ACSM.

## Experimental Approach

**Fig. 2:**
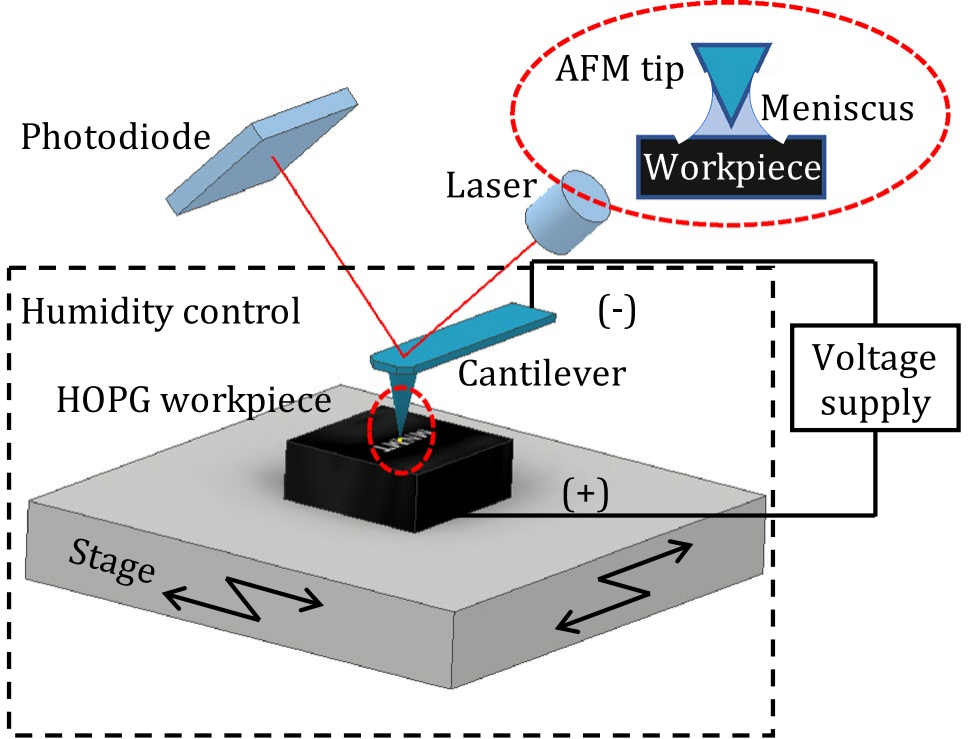
Schematic diagram of the AFM-based electrochemical etching apparatus with an RH-controlled environment

Figure 2 shows the schematic diagram of an AFM-based electrochemical etching apparatus with relative humidity (RH)-controlled environment. Experiments were performed under ambient conditions with a commercial atomic force microscope (MFP-3D Olympus, Asylum Research), which enables precise* x*-*y*-*z*-directional movements. A Si cantilever was used with an overall metallic coating (PtIr_5_) on both sides (PPP-EFM probe, Nanosensor). The tip side coating enhances the conductivity of the tip and allows electrical contacts, and the opposite side coating enhances the laser reflex. The PtIr_5_ coating was an approximately 25-nm-thick double layer of chromium and platinum–iridium on both sides of the cantilever. The tip curvature radius was better than 25 nm. The force constant and resonance frequency of the cantilevers used were approximately 3.74 ± 0.39 N/m averaged over ten different probes calculated using Sader’s method [27] and 75 kHz, respectively. A commercial HOPG was used as a workpiece, and the surface was cleaned by s tripping away several layers before machining using the conventional sticky tape method. The etching voltage could be applied to the AFM tip and HOPG workpiece using AFM controller electronics with a voltage range of − 10 to 10. The applied voltage on the HOPG workpiece was always positive relative to the AFM tip, and the workpiece voltage was monitored by an oscilloscope (Hitachi V-1560). The experiments were conducted at an ambient temperature, approximately 20 ± 1 ℃, with a controlled high RH of 75% in the machining area. RH was controlled by supplying nitrogen gas, which flew through a 1 M saturated sodium chloride (NaCl) aqueous solution to the AFM cell and was continuously monitored by a humidity sensor (HIH-4000 Series, Honeywell).

When an AFM tip was positioned close enough to a sample surface in air, a tiny water meniscus, as shown in the red oval in Fig. 2, was formed in the narrow gap between the tip and sample surface due to a capillary effect. The stability of the water meniscus can be enhanced in humid surroundings. The water meniscus provided the oxygen species (mostly OH^−^) needed to oxidize the workpiece. It also confined the chemical reactions spatially in the nanoscale area by focusing the current density within the water meniscus. Electrochemical etching was performed under the contact mode of the AFM, and the fabrication results were measured by the contact mode. All etching experiments were performed using an applied force of 0.4 µN corresponding to an applied voltage on the AFM tip of 4 V.

## Results and Discussion

### Nano-hole Etching

**Fig. 3:**
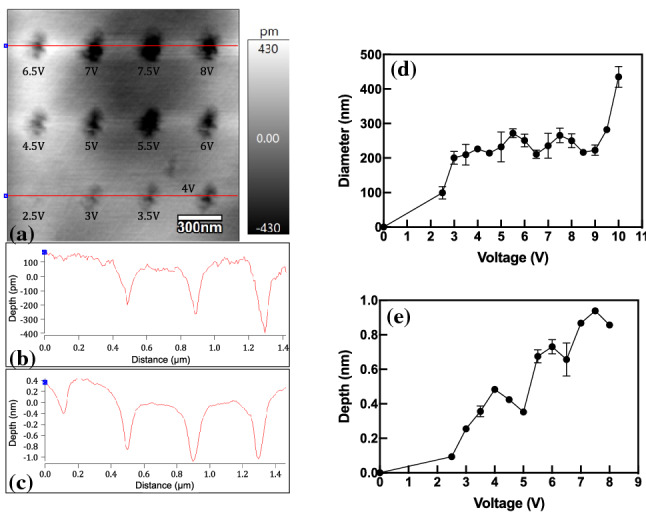
Nano-holes etched with a single-atomic-layer precision: **a** Nano-holes etched with applied potentials of 2.5–8 V and cross-sectional shapes of etched nano-holes with voltages of **b** 2.5–4 V and **c** 6.5–8 V. **d** Diameters and **e** depths of etched nano-holes with applied voltages of 2.5–10 V. The etching duration was constant 20 s

Figure 3a shows the nano-holes etched with the applied potentials from 2.5 to 8 V, and the images were measured by the contact mode of the AFM. The depths of the nano-holes were less than 500 pm with the applied voltages of 2.5 V to 4 V, as shown in Fig. 3b. It is considered that the etching process occurred at a single-atomic-layer precision. However, the depths of the nano-holes were increased to 1 nm with the applied voltages of 6.5 to 8 V, as shown in Fig. 3c, indicating the material removal depth of approximately three atomic layers. Figure 3d and e show the diameters and depths of etched nano-holes with different applied voltages, respectively. The diameter only slightly increased until 9 V, but a sharp increase could be observed between 9 and 10 V. In conventional pulse electrochemical machining, the diameter of a micro-hole is obviously increased by increasing the applied voltage due to the widened working gap width between the tool electrode and workpiece [28]. In this study, because the electrochemical etching was limited in a nanoscale space by the water meniscus, although the voltage was increased, the diameter of the nano-hole was only slightly increased, as shown in Fig. 3d. The image of a water meniscus formed between an AFM tip and a surface has already been measured using environmental scanning electron microscopy [29]. The heights of the meniscus (in the absence of bias) were 100 to 1200 nm with a high RH of 70%–99%, and the diameters of the water meniscus were roughly 300–2700 nm with RH of 40%, 60%, and 99%. When measuring at a high RH, the height of the meniscus is orders of magnitude larger than calculated using either the Kelvin equation or Monte Carlo modeling. Conversely, when the applied voltage was higher than 9 V, Fig. 3d shows that the diameters of the nano-holes were significantly increased, and the edges of nano-holes became irregular. Arguably, the etched debris from the HOPG surface was deposited on the AFM tip due to a large amount of material removal volume with a high applied voltage. Under the influence of contamination from the tip or substrate, the surface water tension of the AFM tip could have been altered [30]. This condition explains the increased diameter of the etched nano-hole at high voltages. Previous studies report that at a high voltage, inconsistent dimensions and tip damage possibly occurred due to mechanical damage and deposited carbon [31]. In this study, when a voltage higher than 10 V was applied on the HOPG surface, the workpiece surface was severely damaged, which is in agreement with the previous research results.

Figure 3e shows that the depth of nano-holes increased with increasing voltage. The depth of each hole was measured three times with an angle of 120° between two adjacent measurements. Jiang and Guo [26] found that no nanostructures were obtained with the ramp voltage from 2 to 1 V, but at higher voltages, convex structures were generated. Our results, as shown in Fig. 3, are in agreement with their study results as no hole was observed with the applied voltage of 2.5 V. The depth of concave holes in their study increased with increasing voltage, which also agrees with our results shown in Fig. 3d.

**Fig. 4:**
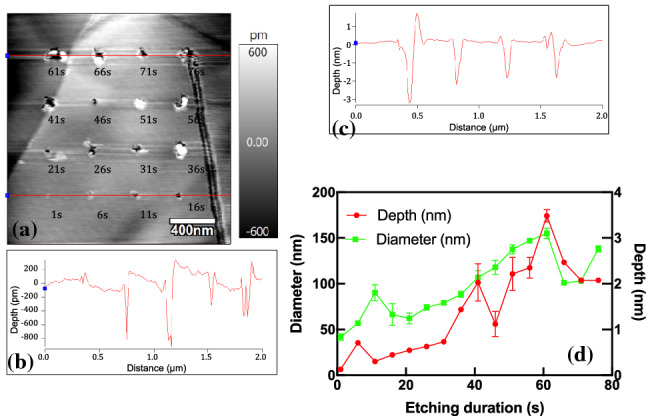
**a** Etched nano-holes with etching durations of 1–76 s and cross-sectional shapes of nano-holes with durations of **b** 1–16 s and **c** 61–76 s. **d** Depths and diameters of the etched nano-holes. The applied tip bias was 4 V

Figure 4a shows the etched nano-holes with different etching durations. Figure 4b shows the line profiles depicting the depths of nano-holes. The depths were less than 800 pm with durations of 1 to 16 s, indicating material removal in one or two atomic layers (Fig. 4b). Moreover, the depths were increased to 2 nm with etching durations of 61–76 s, as shown in Fig. 4c. Figure 4d shows the depths and diameters of etched nano-holes with etching durations from 1 to 76 s. The depth and diameter were increased with increasing etching duration. Nano-holes could be observed even when the etching duration was as long as 76 s, as shown in Fig. 4a. Moreover, some debris can be observed in Fig. 4a when the etching duration was longer than 36 s. The anode and cathode reaction in the electrochemical etching of HOPG can be described by the following reactions [32]:

1 $$ \text{Anode}\;\text{C}  + n{\text{H}}_{2} {\text{O}} \to {\text{CO}}_{n} + 2n{\text{H}}^{ + } + 2n{\text{e}}^{ - } (n = 1\;or\;2) $$

2 $$ \text{Cathode}\;2{\text{H}}_{2} {\text{O}} + 2{\text{e}}^{ - } \to {\text{H}}_{2} + 2{\text{OH}}^{ - } $$

Hence, the carbon would be converted to CO or CO_2_ gas, and there should be no debris formed in the etching area. The debris in Fig. 4a could be attributable to the reaction intermediates before the generation of CO or CO_2_ gas. The generation of reaction intermediates can be described by the following reactions [33]:

3 $$ \text{C}_{x} + \text{H}_{2} \text{O} \to \text{C}_{x} - \text{OH} + \text{H}^{ + } + \text{e}^{ - } $$

4 $$ \text{C}_{x} - \text{OH} \to \text{C}_{x} - \text{O} +  \text{H}^{ + } + \text{e}^{ - } $$

5 $$  \text{C}_{x} -  \text{O} + \text{H}H_{2} \text{O} \to \text{C}C_{x} \text{COOH} + \text{H}^{ + } + \text{e}^{ - } $$

6 $$  \text{C}_{x - 1} \text{COOH} \to \text{C}_{x - 1} + \text{CO}_{2} + \text{H}^{ + } + \text{e}^{ - } $$

where C_*x*_ denotes carbon atoms in the lattice. The intermediates C_*x*_–OH, C_*x*_–O, and C_*x*−1_–COOH form the debris, as shown in Fig. 4a. The high applied voltage and RH are of critical importance for the complete gasification of graphite, resulting in the formation of nano-holes and trenches. However, when the etching was conducted under mild conditions, i.e., with voltage less than 8 V and RH less than 40%, protruded oxide features were formed due to the incomplete gasification of graphite [34]. It is considered that the applied voltage of 4 V was not high enough for complete gasification, resulting in the generation of debris shown in Fig. 4a, especially with a long etching duration. Many papers reported that protruded oxide features could be formed on the HOPG surface using the electrochemical method [26, 35, 36]. However, a single protruded nano-dot could not be obtained in this study. The high RH of 75% might be one of the dominant reasons.

In addition, anode reaction (1) shows that CO and CO_2_ can be generated in the electrochemical etching of HOPG. The etching process can be described by the following reactions:

7 $$\text{C + H}_{2} \text{O(liq.)} + 175.32\;\text{kJ} \cdot \text{mol}^{ - 1} = \text{CO(gas) + H}_{2} \text{(gas)}$$

8 $$ C + 2H_{2} O(liq.) + 178.17\;\text{kJ} \cdot mol^{ - 1} = CO_{2} (gas) + 2H_{2} (gas) $$

Reaction (7) is considered the dominant reaction because reaction (8) requires two water molecules and high energy [25]. Therefore, more CO gases were generated in the electrochemical etching of HOPG than CO_2_. Based on the etching results of the nano-holes, more complicated patterns, such as nano-lines, need to be etched.

### Nano-line Etching

**Fig. 5:**
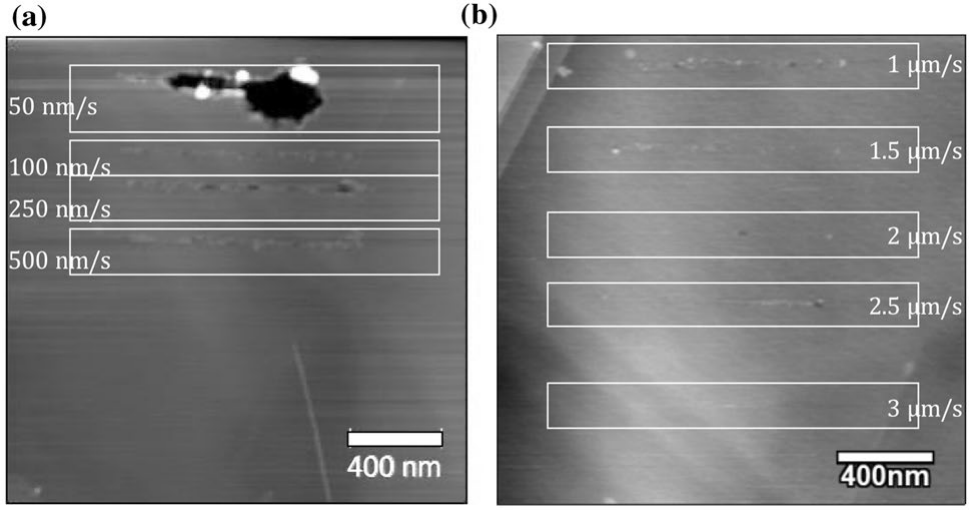
**a** Nano-lines etched with scan speeds of 50 to 500 nm/s. **b** Nano-lines etched with scan speeds of 1 to 3 µm/s. The applied tip bias is 4 V. Both images are of the same size

Figure 5a and b show the nano-lines etched with scan speeds of 50nm/s to 3 µm/s. The widths of the etched lines were reduced with increasing scan speed due to the shorter etching duration. A line with a scan speed of 3 µm/s was not obtained. This is because with higher tip velocity, very few points of contact occur between the tip and substrate for enough atoms to get displaced. Because of the incomplete gasification, some debris was also observed in the etched lines. Moreover, the accuracy of lines was significantly influenced by the wear of the AFM tip.

**Fig. 6:**
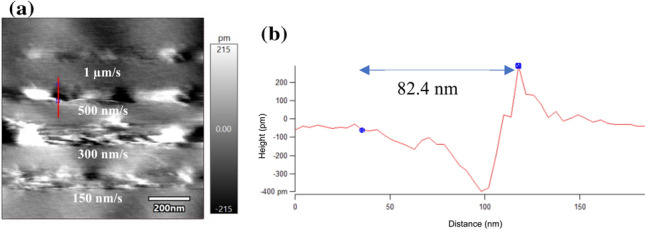
**a** Nano-lines etched with a worn AFM tip. The scan speeds were 150, 300, 500nm/s, and 1 µm/s, and the applied tip bias was 4 V. **b** The line profile showing the trench and its width of 82.4 nm is shown with the* blue line*

Figure 6a shows the nano-lines etched with a worn AFM tip with scan speeds of 150, 300, 500nm/s, and 1 µm/s. The width of the etched line was significantly increased due to the wear of the AFM tip. With a scan speed of 500 nm/s, the width of the etched line shown in Fig. 5(a) was measured as 47.3 nm; however, it was increased to 82.4 nm with a worn tip, as shown in Fig. 6(b).

**Fig. 7:**
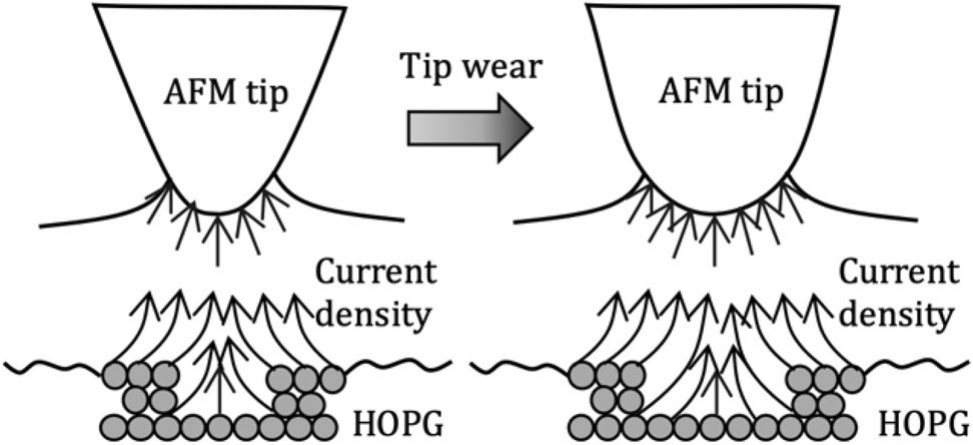
Schematic diagram of the influence of the tip wear on the etching current density

The tip curvature radius increased with the worn AFM tip due to its enlarged water meniscus. Therefore, the current density flowing through the gap between the AFM tip and HOPG surface was increased as the gap resistance was decreased. Moreover, the current density localization effect of the water meniscus was reduced when the tip curvature radius was increased, as shown in Fig. 7. The diameter of the water meniscus was increased with a worn AFM tip, and the effective area for flowing current was increased between the AFM tip and HOPG surface. Hence, electrochemical etching could occur in a wider area, resulting in an increase in the width of the etched nano-line. In conventional electrochemical machining with a tool electrode immersed in an electrolyte, the stray current would flow through the side surface of the tool electrode [28, 37]. Figure 7 also shows that the influence of the stray current density was decreased significantly due to the localization effect of current density by the water meniscus.

This condition contributed to the diameter of the etched nano-hole, which slightly increased with increasing applied voltage, as shown in Fig. 3d. The water meniscus significantly reduced the influence of stray current. Hence, the effect of stray current on the diameter of the etched nano-holes was negligible with increasing applied voltage.

### Intricate Pattern Etching

**Fig. 8:**
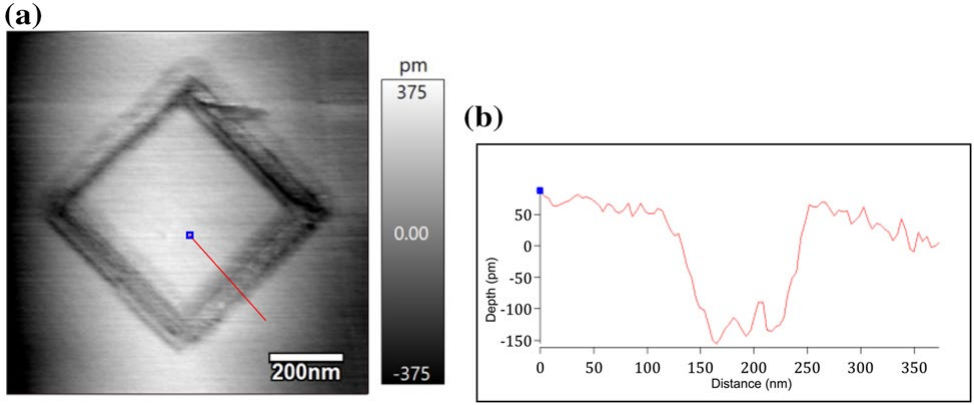
**a** Nano-square etched in a 5 µm × 5 µm area with a scan speed of 1 µm/s and applied tip bias of 4 V. **b** Line profile showing the depth of the trench formed on one side of the square

**Fig. 9:**
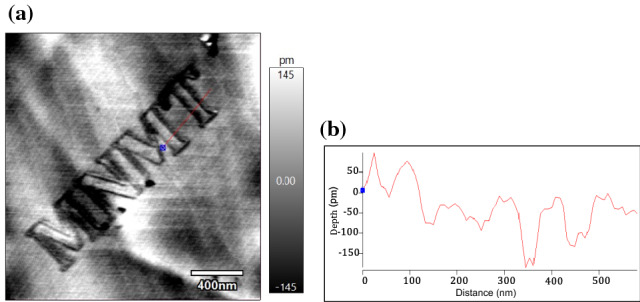
**a** Etched “MNMT” letters in the surface area of 2  µm × 2 µm with a scan speed of 1 µm/s and applied tip bias of 4 V. **b** Line profile of the portion shown in the figure

Considering the above results from nano-holes and nano-lines, a nano-square in a 5 × 5 µm area with a scan speed of 1 µm/s was drawn on the surface, as shown in Fig. 8a. The measured surface showed a trench feature with a depth of 250 pm (Fig. 8b), which indicates a single-atomic-layer etching of the HOPG surface. Furthermore, Fig. 9a and b show the etched “MNMT” letters in the HOPG area of 2 µm × 2 µm close to 335 pm, which also indicates single-atomic-layer precision. Hence, the AFM-based electrochemical etching was verified as an effective method for the intricate pattern etching toward single-atomic-layer precision.

## Conclusions

This study focuses on the single-atomic-layer lithography on the HOPG surface using the AFM-based electrochemical etching technique. The experimental results show that single-atomic-layer precision can be achieved on the HOPG surface for different intricate patterns. This study indicates that AFM-based electrochemical etching is not only effective for nanomanufacturing but also a promising technique for ACSM. The following conclusions can be drawn.

1. The diameter of etched nano-holes slightly increases with increasing applied voltage mainly due to the localization of current density in a small nanoscale space by the water meniscus. The depth of etched nano-holes increases with increasing applied voltage, which is closely related to the electrostatic force between the AFM tip apex and HOPG surface due to the applied voltage.
2. The applied voltage on the HOPG surface has a more dominant effect on the nano-hole etching than the etching duration. The HOPG surface would be damaged with a significantly high voltage, typically higher than 10 V.
3. The width of etched nano-lines increases when the AFM tip is worn because the diameter of the water meniscus is increased between the AFM tip apex and HOPG surface due to the increased tip curvature radius. This condition results in less localization ability of current density from the water meniscus.
4. Intricate patterns, such as “MNMT” letters, are etched successfully toward single-atomic-layer precision, which indicates that AFM-based electrochemical etching can be a promising method for ACSM.
